# Too perfect to be good? An investigation of magicians’ Too Perfect Theory

**DOI:** 10.7717/peerj.13449

**Published:** 2022-05-30

**Authors:** Alice Pailhès, Kole Lee, Gustav Kuhn

**Affiliations:** Department of Psychology, Goldsmiths College, University of London, London, United Kingdom

**Keywords:** Magic tricks, Too-perfect theory, Problem-solving, Explanation, Alternative solution

## Abstract

The “Too Perfect Theory” states that if a trick is too perfect, it might paradoxically become less impressive, or give away its secret method. This theory suggests that an increased impossibility results in a less magical effect. The Too Perfect Theory is often applied to magic effects, but it conflicts with recent scientific investigations showing that participants’ level of enjoyment of a magic performance is positively related to their perceived impossibility of the trick. The current article investigated whether an imperfect magic performance is more impressive than a perfect one. Across two experiments, we studied whether participants enjoy a performance more if the effect is not perfect. We also examined the different types of explanations people give to these two types of performances. The results showed that participants enjoyed a perfect performance more than an imperfect one. However, consistently with the Too Perfect Theory, participants watching the perfect performance also discovered the correct method behind the magic trick more frequently and believed the performance was staged more often. Moreover, participants’ method explanation significantly impacted their reports about the performance.

## Introduction

We usually find things to be perfect when they are devoid of flaws, and this principle applies to many artistic performances. We do not enjoy seeing a juggler dropping their balls, an actor forgetting their lines or a musician playing the wrong chords. However, in the world of magic, perfection itself can be a flaw, and this has led to the suggestion that some magic tricks are too impossible. Most people who choose to watch a magic show know that magic is not real. Even though the experience itself feels real, on a rational level, spectators know that magic tricks are not achieved by supernatural means (Leddington, 2016). Indeed, it is this conflict in beliefs that is central to the art of magic (Kuhn, 2019; Lamont, 2013). Because of this, spectators often leave a magic show with some rational idea of the means by which the thing they have witnessed was achieved. In many instances they may come up with a wrong, yet plausible solution. For example, imagine a magic trick in which the magician claims to be using mind reading powers to deduce the name of a freely chosen playing card. There are lots of different potential explanations for this effect, and people might think that a magician used a tricked deck to control the spectator’s card and correctly guess its identity, even when this is not the case. In *The Magic Way,* Tamariz highlights the importance of eliminating such plausible, yet wrong solutions, as they inadvertently reduce the cognitive conflict that magic elicits and as such reduce the magical experience itself (Tamariz, 1988). Some tricks are simply too perfect and they end up permitting a unique possible method, the correct solution. Therefore, it appears that magicians should probably eliminate, for the audience, the most plausible method to make it harder for them to guess the actual method—thus making the performance more ‘magical’. Introducing imperfections in a performance might bring uncertainty in the spectators’ mind, leading them away from the secret method. For instance, getting the colour and number of the spectator’s card right, but with the wrong suit (*e.g.*, three of hearts instead of three of diamonds) might rule out the possibility that the magician used a forcing technique to control the spectator’s card choice (Pailhès, Rensink & Kuhn, 2020; Pailhès & Kuhn, 2021).

Rick Johnsson proposed the Too Perfect Theory (Johnsson, 1970) which states that, if a trick is too perfect, it might paradoxically become less impressive, or give away its secret method and ruin the performance. Johnsson proposed two premises to explain the theory: (1) in the twentieth century people no longer attribute the magician with supernatural powers, and (2) to a rational person, the unknown is unacceptable. Johnsson argues that rapid scientific and technological advances combined with the frequent public exposure of magic secrets has resulted in modern audiences giving more credit to the magician’s skills rather than their supernatural ‘powers’. Johnsson further suggests that, since the beginning of time, humans have been searching for causal explanations behind the mysteries that surrounds them. These two premises explain why spectators feel the need to explain how the trick is done (see Jay, 2016). However, magic relies on people not knowing the true cause of the effect (Kelley, 1980), and a good performance should prevent the audience from correctly deducing, or even guessing, the secret method (Tamariz, 1988). Thus, according to the Too Perfect Theory, if a trick is so perfect that it no longer leaves room for any explanation than the correct one, it becomes less enjoyable. In other words, the theory suggests that an increased perceived perfection results in a less magical effect. As some authors point out (Carney, 2001; Twose, 2020), in magic, perfection might make a solution obvious, thereby undercutting the perceived impossibility of the performance. For instance, a trick seen on television that appears ‘too perfect—such as a magician correctly guessing all five words that a spectator is thinking of—might be explained in terms of camera tricks, or stooging. However, if the performer introduces an error and misses one of the words, the performance might be more likely to be interpreted as ‘true’ mind reading skills, leaving the audience therefore being more impressed as well.

The “Too Perfect Theory” is often applied to mentalism effects, but the theory conflicts with recent scientific investigations into people’s enjoyment of magic. Bagienski & Kuhn (unpublished data) used a trick in which the magician balanced different objects by stacking them on top of each other and making the trick increasingly impossible, and participants were asked to rate the level of impossibility and their enjoyment at each stage of the effect. Results showed that participants’ level of enjoyment of the performance was positively related to their perceived impossibility of the trick (*i.e.,* as the performance became more and more impossible). We have recently also shown that people’s enjoyment of a trick is positively correlated with their perceived level of impossibility (Kuhn, Pailhès, Jay & Lukian, unpublished data). However, crucially in this study, participants watched different versions of a trick in which the magician predicted the number named by a spectator. Participants enjoyed a performance just as much when the volunteer chose a number between 1 and 4, compared to when the choice was not restricted. This latter effect represents a magic trick that is pretty much perfect, and yet, it was not enjoyed less than the less perfect one.

The Too Perfect theory has been heavily debated in the magic literature. For instance, well-respected magician Tommy Wonder called this theory “one of the worst concepts to appear in magic in a very long time” (Wonder, 1996, p. 2), stating that it is not only wrong but also highly detrimental to the art. Darwin Ortiz also discusses the Too Perfect Theory stating that he does not entirely agree with it, and states that every time he brings it up during a conversation, it is attacked (Ortiz, 2006). Likewise, Tom Stone (Jay, 2021) explains that the theory is incomplete as it does not make firm predictions about the success of an effect. As such the Too Perfect Theory does not make explicit which tricks would benefit from being flawed.

Moreover, magician Dominic Twose (Twose, 2020) proposes that the Too-Perfect theory is misnamed and should be called the Too Obvious theory. Accordingly, if the spectator guesses the correct method of a trick, it is because the method is the obvious one, rather than too perfect. Likewise, John Carney encourages us to think of this principle as the ‘What-Else? Theory’ or the ‘One Solution theory’ (Carney, 2001), as the problem lies in providing alternate solutions than the secret one to the spectators. Rather than findings ways to make a trick less ‘perfect’, the solution would be to offer a plausible alternative explanation to the trick.

The Too-Perfect Theory also relates to another magic theory, known as the theory of False Solution (Kuhn et al., 2014; Lamont & Wiseman, 2005; Tamariz, 1988). Recent research (Thomas, Didierjean & Kuhn, 2018) shows that exposure to an obvious false solution (*e.g.*, a performer hiding a card in the palm of his hand to secretly transfer it to his pocket) prevents participants from finding the real method (*e.g.*, the magician used a duplicate card), even when the magician proves that this false solution is impossible (*e.g.*, showing that their hand is empty). The authors explain this effect with the *peripheral representation hypothesis*, whereby a false solution activates a peripheral representation of the problem, which remains active even after the erroneous solution has been ruled out. If the magician entices the spectator to come up with an erroneous explanation, the peripheral part of the false representation inhibits the discovery of the correct solution, even if this solution is ruled out later on (Thomas & Didierjean, 2016).

Over the years, the Too Perfect Theory has been debated, discussed, embraced and attacked (Racherbaumer, 2001). However, to our knowledge, no scientific investigations has tested it. Still, many magicians seem to believe that introducing imperfection into their tricks can make those tricks better, as their method would thereby become less obvious. The current article aims to study one application of this idea that is especially common in mentalism—that introducing imperfection in the sense of making a trick less reliable might increase participants’ enjoyment and perceived impossibility of this trick. In our first experiment, we investigate whether participants enjoy a performance more when the magician’s mind-reading is slightly off target than when it is perfect. Our second experiment follows Carney’s (2001) suggestion and further examined the different types of explanation people give to these two types of performances.

### Experiment 1

The aim of the first experiment was to evaluate whether a perfect performance was less impressive and enjoyed than an imperfect one. We sought to investigate whether a performance in which the magician succeeded in all his effects (*i.e.,* correctly guessing the colour, suit and number of a freely chosen card) produces lesser appreciation of the trick than one in which the magician provides an imperfect effect (*i.e.,* correctly guessing the colour and suit but not the number of the card). Based on the Too Perfect Theory, we predicted that participants would find the performance more impressive and enjoyable when it was imperfect.

### Methods

### Participants

A total of 123 participants (40 female) between 18 and 56 years old (*M* = 24, *SD* = 7.17), who were all recruited on Prolific, took part in the experiment. Goldsmiths Psychology Department provided ethical approval for all experiments. For all experiments, participants provided written informed consent before participating. We confirm that for the two experiments we reported all measures, conditions, data exclusions and how we determined our sample sizes.

The sample size was calculated thanks to a power analysis for a *t*-test for independent means, with *d* = .5 (medium effect size), *α* = .05, and a power of .80. We based our effect size estimation on the magic literature and by looking for an effect we estimated worth-finding. The output of the calculation was 102 participants.

### Procedure

The survey was implemented online *via* Prolific. After reading the information page on which participants were told they were going to watch a short video of a magic trick and General Data Protection Regulations, participants confirmed they accepted to take part in the study and signed the consent form. Then, we displayed one of the two videos in which a magician performed the same trick with two different outcomes and randomly attributed each participant to one of two experimental conditions: Perfect performance or Imperfect performance. In all conditions, the magician asked a spectator (a confederate) to pick a card out of a deck (the 9 of Diamonds), look at it and think about it. The magician then instructed the spectator to think of the card, and he asked her to think about whether it was a red or black card. He then proceeded to correctly name the colour (red). The magician then asked the spectator to think of the suit, and again he correctly named it (Diamond). Finally, he asked the spectator to imagine the number and then either guessed that the card was a 9 of Diamonds (Perfect performance) or a 6 of Diamonds (Imperfect performance).

After watching one of these videos, participants had to state on scales from 0 (not at all) to 100 (very much) how impressed they were by the performance, how difficult they thought it was to perform, how much they enjoyed the performance, how surprised they were about the outcome of the performance. Based on the Too Perfect Theory, we expected all these measures to be significantly higher in the Imperfect rather than in the Perfect performance. We also asked participants to estimate the likelihood of the performance succeeding (from 0 to 100%) as a mean to measure perceived impossibility, and expected that participants would report higher means in the Perfect rather than Imperfect condition.

## Results and discussion

Overall, participants were moderately impressed (*M* = 52.2, *SD* = 27.2) and surprised (*M* = 49.7, *SD* = 28.4). They also felt that the performance was moderately difficult to perform (*M* = 55.4, *SD* = 26), reported medium levels of enjoyment (*M* = 50.3, *SD* = 28.2) and estimated a moderate likelihood of the performance succeeding (*M* = 56.4 *SD* = 24.2).

**Figure 1:**
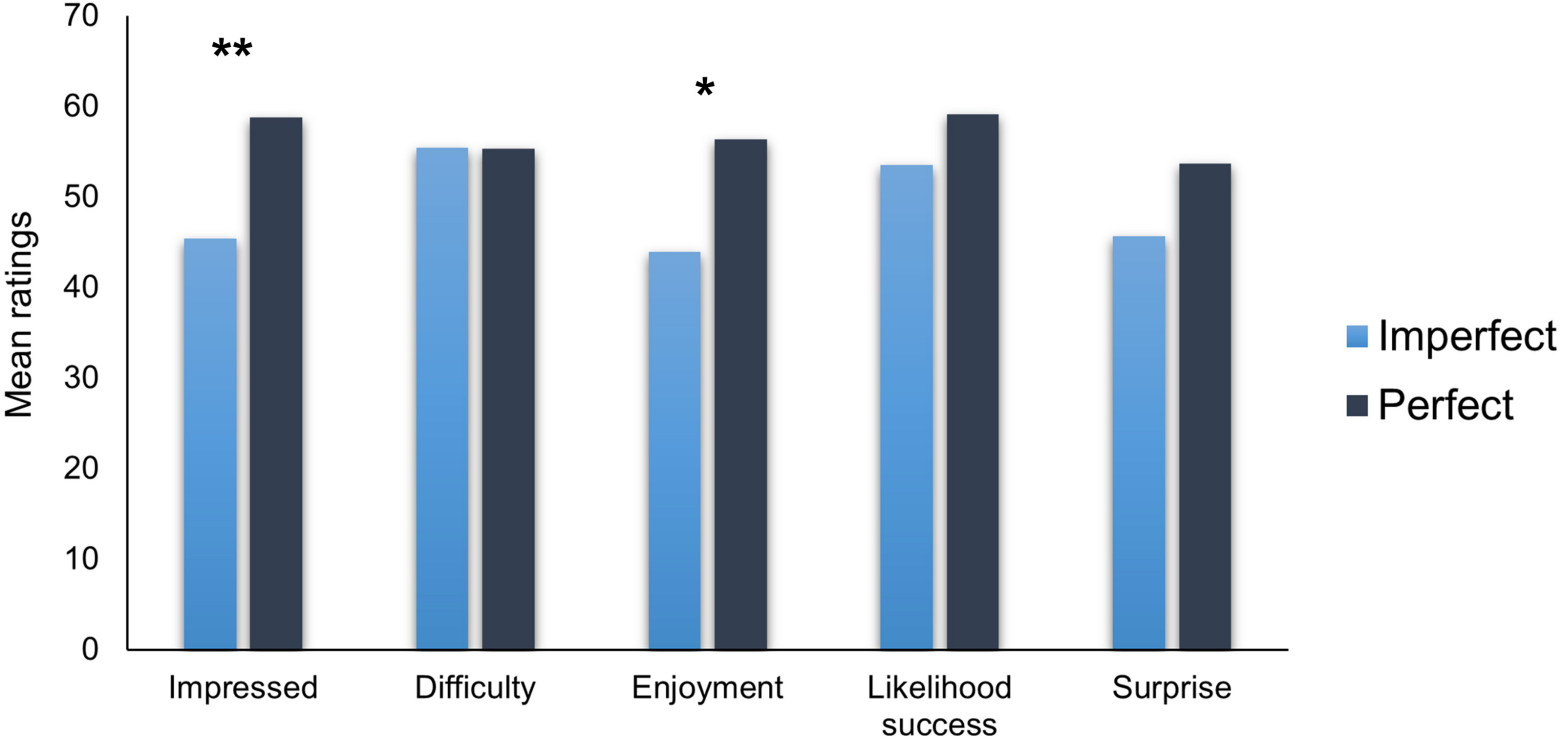
Mean ratings of the magic trick depending on the type of performance. Error bars display standard errors. *p < .05, **<.01.

As the data were not normally distributed, we used Mann–Whitney non-parametric tests. Looking at the two types of performance (Fig. 1), participants felt significantly more impressed in the Perfect performance than in the Imperfect one (*W* = 1,264, *p* = .005, *r*_*rb*_ = −.298) and enjoyed the Perfect performance more than the Imperfect one (*W* = 1,359, *p* = .021, *r*_*rb*_ = −.245). This goes against our predictions, suggesting that the performance in which the magician correctly guessed all the features of the spectator’s chosen card was more impressive and enjoyable than the imperfect performance. These results support John Carney’s (Carney, 2001) view that simply making the trick imperfect does not increase observers’ enjoyment of the performance. Instead, the Too-Perfect Theory might rely more on providing an alternative explanation for the effect.

Looking at the other measures, participants did not report significantly different levels of estimation of the performance’s difficulty (*W* = 1,785, *p* = .941, *r*_*rb*_ = −.008), likelihood of succeeding (*W* = 1,509, *p* = .126, *r*_*rb*_ = −.161) and did not report significantly different levels of surprise (*W* = 1,524, *p* = .148, *r*_*rb*_ = −.153) according to the performance.

Next, we examined the correlations between our five measures. Table 1 shows that all our variables except perceived difficulty and likelihood of success correlate positively with one another. The strongest correlation was between participants’ enjoyment of the performance and how impressed they were by the trick. This confirms recent results about participants’ estimation of a trick impossibility and their appreciation of it (Bagienski & Kuhn, unpublished data; Kuhn et al., unpublished data). There was no significant correlations between participants’ estimation of the trick’s likelihood of success and their perceived difficulty of it. If we estimate that a trick is very likely to succeed, it is reasonable to also think that it is not so difficult to perform.

**Table 1:** Correlation matrix showing Spearman’s correlations between each of the variables.

	**Impressed**	**Difficulty**	**Enjoyment**	**Likelihood success**	**Surprise**
Impressed	–				
Difficulty	.377^***^	–			
Enjoyment	.819^***^	.384^***^	–		
Likelihood success	.427^***^	.037	.427^***^	–	
Surprise	.729^***^	.448^***^	.722^***^	.328^***^	–

**Notes.**

* *p* < .05.

** *p* < .01.

*** *p* < .001.

We kept this first experiment as simple as possible to exclude other possible factors than the performance’s ‘perfection’ that could impact participants’ appreciation of the performance. However, we believe that the current experiment missed an important factor. In the current videos, the magician did not provide any explanation for his imperfection, which might just have been perceived as a failure of the entire effect. Providing an explanation for the imperfection might provide us the opportunity to introduce an alternative solution (*e.g.*, the magician used mind reading skills) than the secret one (*i.e.,* here, the use of a stooge) in the participants’ mind, making them enjoy the performance more.

### Experiment 2

The previous study does not support the Too-Perfect Theory prediction, and the results suggest that people enjoy a trick more when it is devoid of imperfection. In the second experiment, we aimed to address one of the main limitations by including an imperfect performance in which the magician provides an explanation for the mistake that is in line with the intended magical effect. We expected that the justification provides an alternate explanation for the trick that should strengthen the effect, and reduce the chances of people guessing the real method (*i.e.,* use of a stooge/actor).

As the Too-Perfect Theory is thought to induce alternative solutions, we examined the nature of these explanations by asking participants to explain how they thought the trick had been done. We predicted that the imperfect performances would result in a different type of explanations, and that they would reduce the chances of people suspecting the use of a confederate. We also expected participants to appreciate the performance more when the magician provided an explanation with his imperfection.

### Methods

### Participants

A total of 338 participants (146 female) between 18 and 65 years old (*M* = 24, *SD* = 7.17), who were all recruited on Prolific, took part in the experiment. The sample size was calculated thanks to a power analysis for a fixed-effects ANOVA, with *f* = .18 (small to medium effect size), *α* = .05, and a power of .80. We based our estimation of the effect size on the effects of our first experiment and the output of the calculation was 301 participants.

### Procedure

The same procedure as Experiment 1 was used, except that we had three experimental groups. Participants were randomly allocated to either a Perfect Performance, an Imperfect Performance or an Imperfect Performance with an Alternative Solution. The performances for the Perfect Performance as well as the Imperfect Performance were the same as in Experiment 1. However, this time, for the Alternative Solution condition, the video was the same as the Imperfect one, except that at the end the magician justified his mistake by explaining that through mind reading he must had seen the correct number (9) the other way round in his mind, ending up with the 6 of Diamonds.

After watching one of these three videos, participants answered the same questions as in Experiment 1. This time, we also added a qualitative question asking participants to describe how they thought the performance was accomplished.

## Results and discussion

### Performance ratings

Overall, participants were moderately impressed (*M* = 40, *SD* = 26.9) and surprised (*M* = 41.4, *SD* = 29.1). They also felt the performance was moderately difficult to perform (*M* = 51.78, *SD* = 31.3), reported medium levels of enjoyment (*M* = 39.1, *SD* = 30.9) and estimated a moderate likelihood of the performance succeeding (*M* = 47.9 *SD* = 29.1).

**Figure 2:**
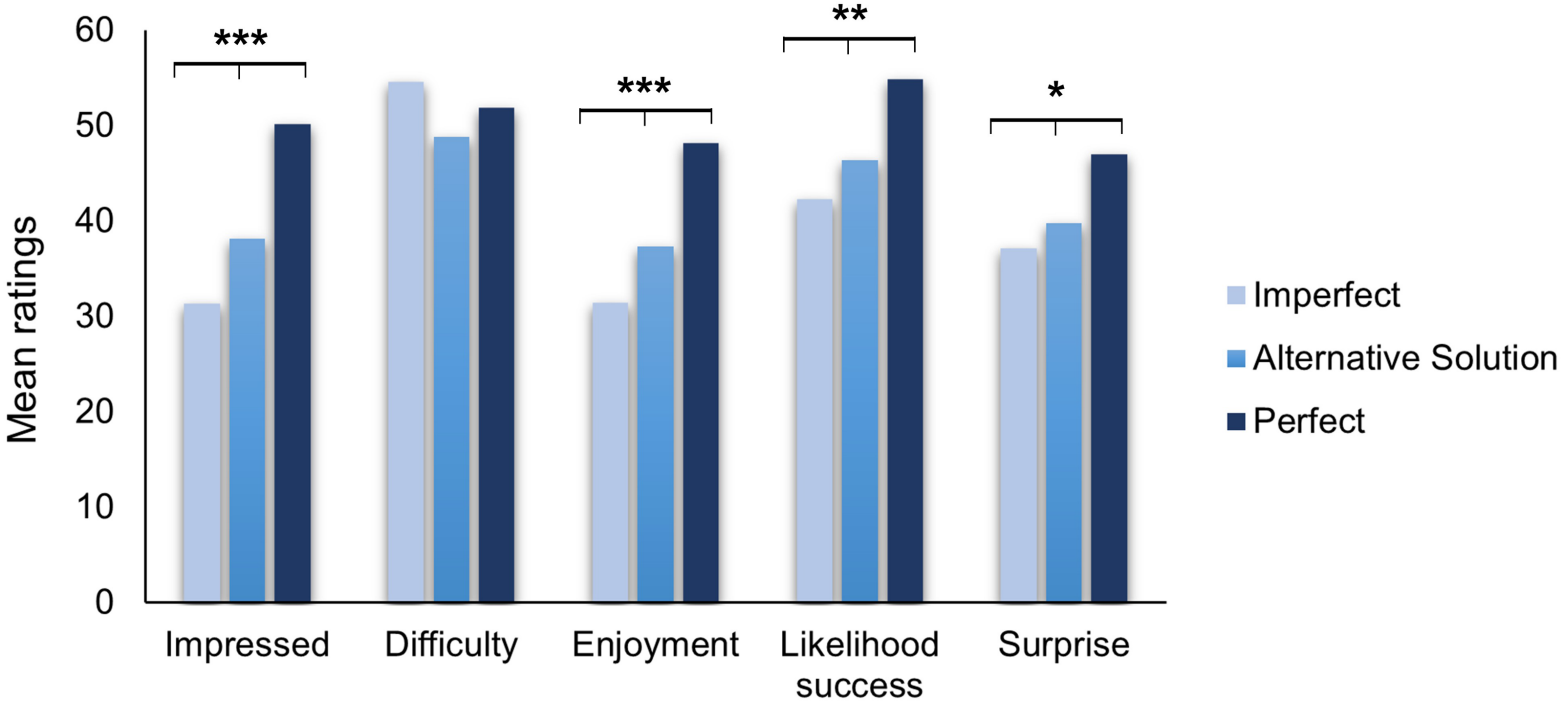
Mean ratings of the magic trick depending on the type of performance. Error bars display standard errors. *p < .05, **<.01, ***<.001.

Then, we conducted Kruskal–Wallis tests and contrast analyses to look at the effect of our types of performances. Participants reported significantly different levels of impression (H(2) = 22.93, *p* < .001) , enjoyment (H(2) = 17.8, *p* < .001) and surprise about the performances (H(2) = 7.01, *p* = .029). The linear contrast analyses showed that participants reported the lower levels for these measures in the Imperfect Performance, higher levels in the Alternative Solution one, and the highest in the Perfect Performance (see Fig. 2). Contrary to our predictions, this suggests that even when the magician provided an alternative solution to justify his imperfect performance, this was still less appreciated than a perfect performance.

Likewise, participants estimated that the likelihood of the trick succeeding was significantly different in the three types of performance (H(2) = 12.28, *p* = .002), contrast analysis showed the same pattern as with the other measures (*t* (337) = 3.63, *p* < .001, Fig. 2). However, participants did not report significantly different estimations of the level of difficulty for the performances (H(2) = 1.87, *p* = .393), replicating results from our first experiment. This suggests that making a mistake during the performance did not affect the audience’s perception of the difficulty of the trick.

Next, we looked at the correlations between our five variables. Table 2 shows that all variables significantly correlated with each other, replicating results from Experiment 1. However, contrary to Experiment 1, this time the perceived difficulty of the trick also correlated with participants’ estimations of the likelihood of success for the trick. This correlation disappeared (*r*_*s*_ =.127, *p* = .058) when we focus on the Perfect and Imperfect performances—the ones used in our first experiment. This suggests that participants associated the trick’s difficulty with its likelihood of success only when they watched a performance that provides an alternative solution.

**Table 2:** Correlation matrix showing Spearman’s correlations between each of the variables.

	**Impressed**	**Difficulty**	**Enjoyment**	**Likelihood success**	**Surprise**
Impressed	–				
Difficulty	.640^***^	–			
Enjoyment	.893^***^	.657^***^	–		
Likelihood success	.360^***^	.153^**^	.373^***^	–	
Surprise	.760^***^	.624^***^	.788^***^	.389^***^	–

**Notes.**

* *p* < .05.

** *p* < .01.

*** *p* < .001.

### Method explanation

We next examined the type of explanations that participants provided for how they thought the trick had been done. We expected that participants would be more suspicious of the Perfect performance than the two others. After analysing participants’ explanations, we came up with five main categories to classify each of their statements. We excluded 45 participants from the analyses as they did not provide any explanations to the trick but simply described what they saw in the video or things such as it was ‘very impressive’, ‘I’m surprised’, *etc.* Participants’ reports were coded by two independent blind coders, who initially agreed on 88% of the reports. The remaining reports were categorized by the subsequent coders’ agreement.

The five categories of method explanations were:

**Correct method**: Participants thought the performance was staged or arranged in some way with the help of a confederate, cooperation or video editing (*i.e.,* guessing the correct solution).

**No idea**: Participants reported not knowing how the trick was done.

**Trick**: Participants mentioned mechanisms involving the magician controlling the spectator’s card choice (forcing technique, tricked or marked deck) or having a physical way to know which card the spectator chose (counting the cards, marked deck, using a mirror).

**Psychological/pseudoscientific explanation**: Participants mentioned explanations including mind-reading, reading subtle micro-expressions and body language.

**Luck**: Explanations that were based on luck or guessing.

Overall, participants most frequently came up with psychological/pseudoscientific explanations (34.7%) followed by explanations based on trickery (24.9%), followed by not knowing the method behind the trick (20%), followed by methods based on luck (11.3%) and finally those who correctly guessed it was staged (9.1%).

**Figure 3:**
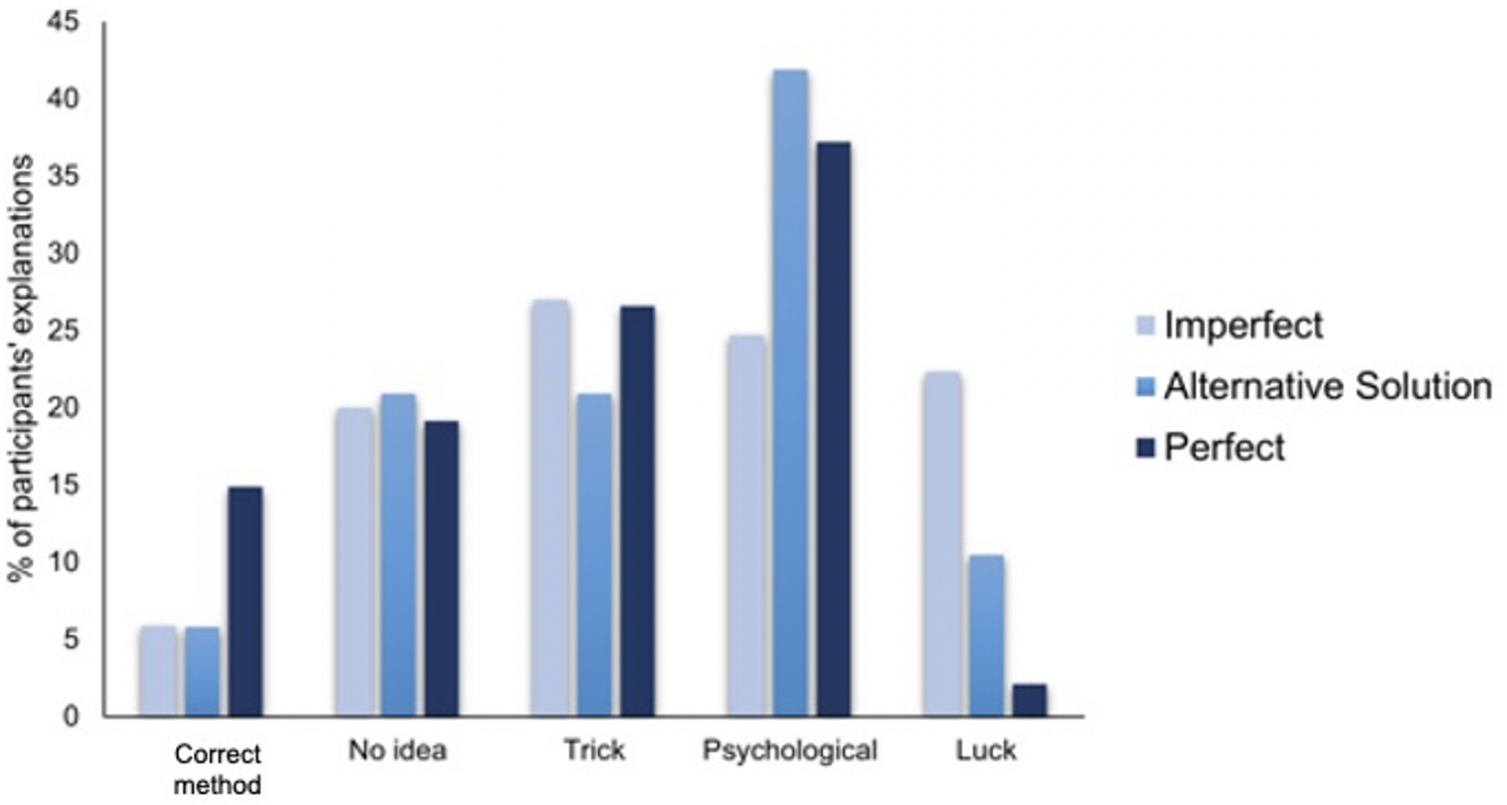
Percentages of participants’ depending on the type of performance and type of method explanation.

A Chi-Square test found a significant difference in participants’ explanations across the three types of performance (*X* ^2^ (8, *N* = 265) = 26.5, *p* < .001, *V* = .223, Fig. 3). Post hoc analyses looking at adjusted residuals showed that more participants than expected provided explanations based on Luck in the Imperfect performance than in the other conditions, and the reverse was true with regards to explanations that guessed the correct method. This confirms our prediction, suggesting that a perfect performance, although more enjoyable, makes spectators more suspicious about the method used and—they are more likely to suspect it was staged. As suggested by the Too-Perfect Theory, a perfect trick resulted in more participants guessing the correct secret method (*i.e.,* use of stooge, ‘fake’ performance) than in the imperfect ones. Moreover, results concerning the Lucky guess suggest that performing an imperfect trick without providing any explanation, led participants to conclude that no “real” method was used and that instead the magician was simply guessing.

### Explanations’ impact on performance ratings

Finally, we looked at how participants’ ratings varied as a function of the explanation they gave. Kruskal-Wallis tests showed that participants’ explanations had a significant impact on how impressed they were by the demonstration (H(4) = 12.3, *p* = .015), how difficult they thought the trick was to perform (H(4) = 15.3, *p* = .004), how much they enjoyed the performance (H(4) = 17.1, *p* = .002), their estimation of the likelihood of the performance’s success (H(4) = 38.1, *p* < .001) and their level of surprise (H(4) = 24.8, *p* < .001).

**Figure 4:**
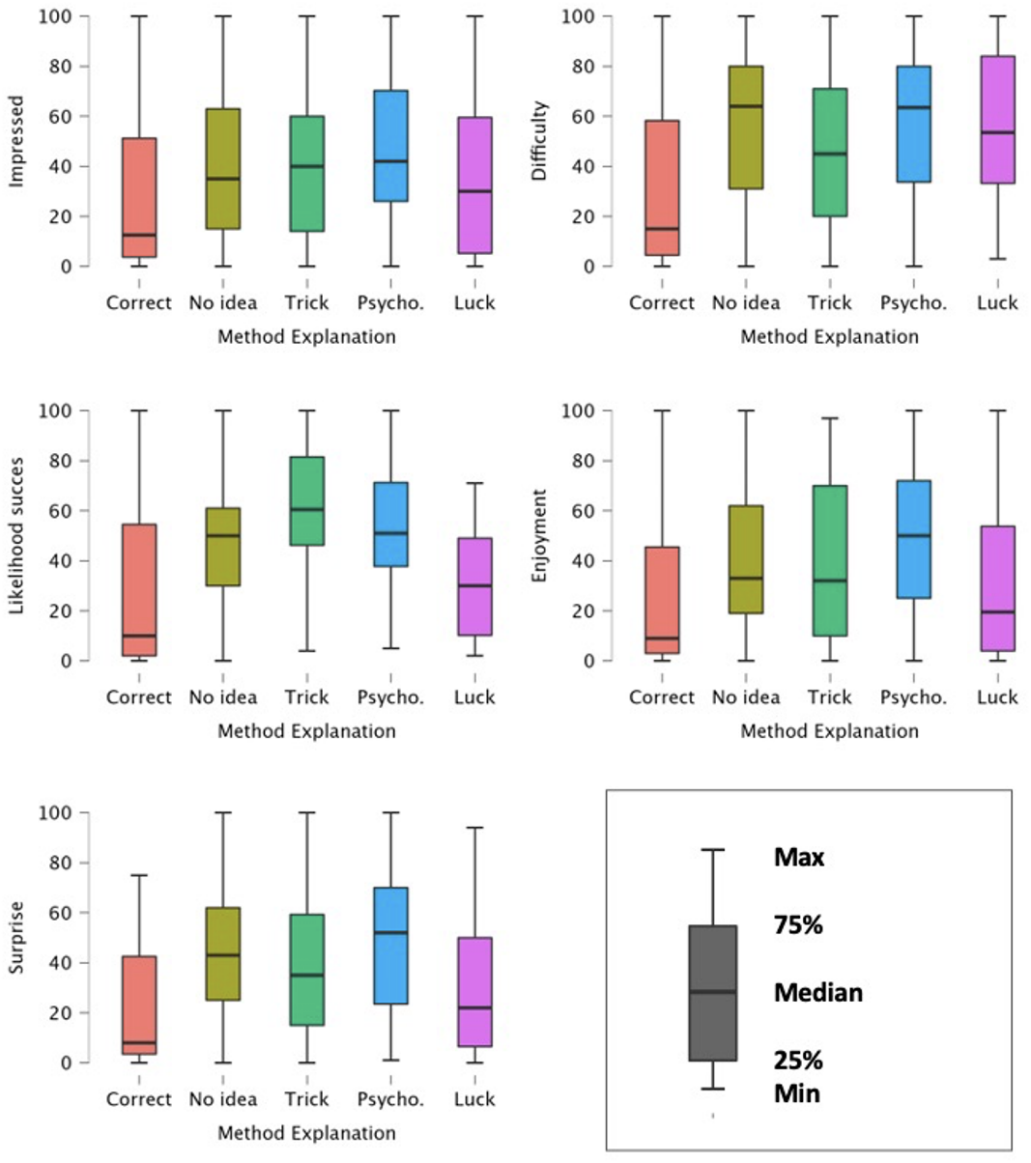
Distribution of participants’ reports about the performance by type of method explanation.

We then proceeded to deviation contrast analyses to inspect the impact of participants’ explanation in more details (see Fig. 4).

### Correct method

The analyses showed that participants who thought the performance was staged—and therefore correctly guessed the secret method—found the performance to be significantly less difficult to perform (*t* (270) = −3.26 *p* = .001) and estimated the likelihood of the performance succeeding significantly lower (*t* (270) = −3.49, *p* < .001). Indeed, using a stooge or video editing might appear as easier to perform than complex sleight of hands or psychological skills. Likewise, participants were significantly less surprised by the demonstration when they thought it was staged (*t* (270) = −2.79, *p* = .006).

#### No idea

Participants who reported not knowing how the performance was accomplished estimated the performance as significantly more difficult than the other participants (*t* (259) = 2.08, *p* = .038). Likewise, these participants also reported higher levels of surprise than the other groups (*t* (260) = 2.13, *p* = .034). This is congruent with the Too-Perfect Theory, suggesting that people who claim not knowing how the trick was performed seem to appreciate it more.

#### Trick

Participants who gave explanations linked to trickery, or physical means, estimated the likelihood of the performance succeeding as significantly higher than the other groups (*t* (260) = 5.18, *p* < .001). This makes sense in that participants in this group often provided method explanation that were based on a range of reliable ways of performing the trick (*e.g.*, using a mirror or a marked deck to see which card the spectator chose, or forcing the spectator’s choice).

#### Psychological/pseudoscientific explanations

As displayed in Fig. 4, contrast analyses showed that participants felt significantly more impressed (*t* (259) = 3.04, *p* = .003), found the trick more difficult (*t* (259) = 2.32, *p* = .021), enjoyed it more (*t* (259) = 3.65, *p* < .001) and were more surprised (*t* (260) = 4.05, *p* < .001) when they gave psychological and pseudoscientific explanations. This confirms our prediction, suggesting that when spectators can come up with an alternative method—or even one which would in principle be usable—they appreciate the performance more. Participants giving pseudoscientific method explanations also estimated the likelihood of the trick succeeding significantly higher than the other participants (*t* (260) = 3.80, *p* < .001).

#### Lucky guess

Finally, participants who thought the performance was accomplished through lucky guess estimated the likelihood of the trick succeeding as significantly lower (*t* (260) = −3.41, *p* < .001) than the other participants.

Overall, the results from this second experiment suggest that people appreciate a perfect performance more than a performance with an imperfection, even when the magician provides an explanation that justifies the mistake. These results are contrary to what we could expect from the Too-Perfect Theory. However, the different performances did lead to different types of method explanation. Participants were more likely to guess the correct method (*i.e.,* staged performance using a stooge) when they watched the perfect performance. Participants who watched an imperfect performance without any alternative explanation were more likely to think that the performance was not based on any ‘real method’ (*i.e.,* the magician relying on luck or simply trying to guess the card). Finally, participants who gave pseudoscientific explanation were the ones who enjoyed the performance the most.

### General discussion

Johnsson’s Too-Perfect-Theory states that “Some tricks, by virtue of their perfection, become imperfect. Conversely, some tricks, by virtue of their imperfections, become perfect”. (p. 50 (Racherbaumer, 2001)). In this article, we sought to investigate these tenets, and examined whether participants appreciate a magic performance more when it contained an ‘imperfection’.

Contrary to what we would expect from the theory, participants preferred a perfect performance to an imperfect one. In Experiment 1 we found that participants preferred a perfect performance in which the magician correctly discovered the card’s colour, suit and number to an imperfect one in which the magician correctly deduced everything except the card’s number—a 6 instead of a 9. Participants rated the perfect performance as more enjoyable and impressive than the imperfect performance. In Experiment 2 we added a further condition in which the magician provided a plausible explanation for the imperfection. This explanation was intended to strengthen the plausibility of the magician reading the spectator’s mind and thus strengthen the effect. Rather than simply misnaming the number, the magician explained that he named the number 6 rather than the 9 because he unintentionally misrepresented the mental image. Magicians often provide these types of explanation, and we anticipated that it should strengthen the effect. However, our results show that participants still preferred a perfect performance.

In Experiment 2 we also examined the type of explanations participants provided for how they thought the trick had been achieved. Our results show that even though an imperfect trick will not necessarily improve how much people enjoy it, the imperfection does change the types of explanations that observers provide. A higher proportion of participants guessed the correct method in a perfect performance than in the imperfect versions—they thought the performance was staged. Adding an imperfection to the trick also increased the proportion of viewers who explained the performance in terms of psychological/pseudoscientific principles. As mentalists often frame their effects around such principles (*e.g.*, using body language or mind reading skills), this is the type of explanation that a performer intends their spectators to endorse. Such type of explanations are often widely removed from actual methods that can be used to perform this kind of trick. Indeed, our subsequent analysis shows that observers who attributed the effect to psychological/pseudoscientific principles appreciated the performance more than the others. This is consistent with the Too Perfect Theory in that it allows people to come up with an alternative method to the correct one, which can potentially result in higher levels of appreciation. However, it is important to note that the quasi-experimental nature of our design prevents us from making any causal attributions, and so we cannot be sure about the direction of this relationship.

Humans perceive the world in terms of causal relationships which means they always need explanations to things that bewilder them (Kuhn, 2019). After observing a magic trick, people often feel compelled to come up with some explanation of how the trick has been achieved, even when the explanation is wrong or implausible (see choice blindness, Johansson et al., 2005). Past research shows that the nature of these explanations can vary depending on situational factors, as well as individual differences. For example, Gronchi & Zemla (2021) demonstrated that when watching a mentalism magic trick, analytical thinkers tend to generate explanation that are more rational (*e.g.*, explanations based on physical props), whilst intuitive thinkers are more likely to generate irrational explanations that accord with the magician’s backstory (*e.g.*, subliminal cues). Likewise, people’s prior beliefs also influence the type of explanations that people provide for such magical effects (Lesaffre et al., 2018; Mohr, Koutrakis & Kuhn, 2014). The nature of such explanations will affect how people experience the magic. If people are led to believe that the performance was staged—as in the present article—observers will be less likely to endorse the intended pseudo-explanation and the magical experience can be reduced. However, our results could also mean that people enjoy a performance more if they believe the magician used pseudoscientific skills to his ends. It is likely that these types of principles elicit more fascination than ‘simple’ sleight-of-hand skills, or methods that rely on physical deception (*e.g.*, marked deck).

Magicians often state that the Too-Perfect Theory does not necessarily refer to situations in which the magic is too good or too strong, but instead refers to obviously impossible circumstances, especially in conjunction with weak presentation. John Carney for instance suggests that magicians should make a solution less obvious rather than make the trick less perfect (Carney, 2001). Accordingly, the theory could be thought of as the ‘One Solution Theory’ or the ‘What-Else? Theory’, with many ways to make this theory work. One alternative could for instance be to use magician Juan Tamariz’s False Solution approach (Tamariz, 1988). For instance, a magician could pretend to palm a card (*i.e.,* hide it under their hand) when in reality they use duplicates of this card to make it appear at different impossible location. Here, mimicking the palming gesture and making the trick ‘less perfect’ in that it could appear as the secret method is revealed because of the magician’s clumsiness results in leading the spectator down the path of a possible solution, which the magician can later make clear is false. The Theory of False Solution seems related to fixation and anchoring effects. Once a possible solution is introduced, it becomes more difficult for the observer to generate an alternative and all generated alternatives will be anchored on the original false solution.

Some magicians claim that enticing observers to endorse psychological explanations of an effect prevents observers from searching for alternatives and that this can results in more enjoyable performances. Mentalism is one of the most popular magic genres (Jay, 2016), and mentalists often frame their performances as psychological demonstrations. Our results demonstrate that participants who endorsed a psychological explanation enjoyed the performance more, and that the imperfection nudged observers towards offering such explanations. Our results also show that a large proportion of our observers felt compelled to endorse a potentially valid method (*e.g.*, marked deck, mirror, counting cards…), but that this reduced their enjoyment of the effect.

Twose (2020) proposes that the Too Perfect Theory should be rather thought of as the ‘Too Obvious Theory’, and that a magician should offer a plausible alternative explanation to the trick rather than making their trick less ‘perfect’. However, our results, contrary to what we could expect based on this approach, showed that participants’ enjoyment of the trick decreased as the method for performing the trick became less obvious. Future research could put the entire focus on the alternate solution rather the imperfections of the trick. One could use ‘perfect’ performances similar to what we used here, but either provide an alternative explanation or not. For instance, we could present our mindreading effect, with a magician asking to participant to freely take a card out of a deck and either having the performer act as if they are trying to read the participant’s microexpressions or directly guessing the correct answer—we predict that participants would be less likely to suspect the use of a one-way deck (all cards are identical) when the magician provides an alternative explanation.

Our studies have two main limitations. Firstly, our results are limited in that we used one specific type of magic trick and the results might not be generalizable to all types of tricks and performers. There are lots of different styles of magical effects, as well as ways in which the same effect can be performed. Magician Tom Stone (Jay, 2021) underlines the fact that the theory is limited to *some tricks*, without defining how those tricks can be recognized. Accordingly, a magician would have to perform a trick first and then judge by the spectators’ reactions whether the trick belongs in the ‘some tricks’ category of the Too Perfect Theory. We focused on a mentalism effect, because this is an area where the Too Perfect Theory is more frequently applied. It is worth-noting that there is some disagreement in the magic community about whether mentalism should be categorized as magic. Although a common assumption is that mentalism is a subset of magic, some magicians and mentalists would disagree with it. It is possible that the Too Perfect Theory will have an impact on other types of tricks than on a mental effect such as the one investigated in this study. However, we feel that the performance, represents a fairly typical context in which the principle would be applied, and thus our results do generalize to a broad range of effect.

On a related note, we investigated one type of imperfection and there are lots of other ways in which such imperfections can be introduced (*e.g.*, missing one word out of several to guess or not finding the spectator’s chosen card in the first place). Further research may focus on some of these other forms of imperfections. It is also possible that our participants simply perceived the imperfection as a failure rather than a slight imperfection. It might therefore be necessary to implement less important imperfections, such as the magician getting the suit slightly wrong rather than the number (*e.g.*, Queen of Hearts instead of Queen of Diamonds). Likewise, we believe that an ‘imperfect’ performance could become more entertaining if the performer explained their error further than what we have done in this study. For instance, the magician could explain that they mistakenly said 6 instead of 9 because when reading the person’s mind they viewed the number upside down, and that mind-reading is a complex task that might take time to know how a person visualize things. However, in its crudest form our results demonstrate that simply adding imperfections to a trick by missing part of a prediction does not automatically improve people’s enjoyment of the trick. To the contrary, people seemed to enjoy these performances less, and they are less impressed.

This article suggests that a magic performance containing an imperfection is less appreciated than a perfect performance. However, aligning with the Too-Perfect Theory from magic literature, people also seem more likely to find the correct—secret—method behind the trick when the performance is perfect than when it is not.

## Supplemental Information

10.7717/peerj.13449/supp-1 Supplemental Information 1 Raw data of experiment 1

10.7717/peerj.13449/supp-2 Supplemental Information 2 Raw data of experiment 2

## Additional Information and Declarations

### Data Availability

The following information was supplied regarding data availability: The raw data are available in the Supplementary Files.
